# Nuciferine Protects Cochlear Hair Cells from Ferroptosis through Inhibiting NCOA4-Mediated Ferritinophagy

**DOI:** 10.3390/antiox13060714

**Published:** 2024-06-12

**Authors:** Xian Gao, Huanyu Mao, Liping Zhao, Xiang Li, Yaqi Liao, Wenyan Li, Huawei Li, Yan Chen

**Affiliations:** 1 ENT Institute and Otorhinolaryngology, Department of Eye & ENT Hospital, Fudan University, Shanghai 200031, China; 2 NHC Key Laboratory of Hearing Medicine, Shanghai 200031, China; 3 Department of Otorhinolaryngology Head and Neck Surgery, The Third People’s Hospital of Hubei Province, Wuhan 430030, China; 4 The Institutes of Brain Science, The Collaborative Innovation Center for Brain Science, Fudan University, Shanghai 200032, China; 5 State Key Laboratory of Medical Neurobiology and MOE Frontiers Center for Brain Science, Fudan University, Shanghai 200031, China

**Keywords:** hearing loss, cochlear hair cells, nuciferine, ferroptosis, ferritinophagy, ototoxicity

## Abstract

Cisplatin is a widely used antineoplastic drug for treating various types of cancers. However, it can cause severe side effects, such as bilateral and irreversible hearing loss, which significantly impacts quality of life. Ferroptosis, an iron-dependent form of programmed cell death, has been implicated in the pathogenesis of cisplatin-induced ototoxicity. Here, we investigated the effects of nuciferine, a natural active ingredient isolated from lotus species, on the ferroptosis of cochlear hair cells. Firstly, our results demonstrated that nuciferine can protect hair cells against RSL3-induced and cisplatin-induced damage. Secondly, nuciferine treatment reduced ferrous iron (Fe^2+^) overload in cochlear hair cells via inhibiting NCOA4-mediated ferritinophagy. Inhibition of ferritinophagy by knocking down *Ncoa4* alleviated cisplatin-induced ototoxicity. Importantly, nuciferine treatment mitigated cochlear hair cell loss and damage to ribbon synapse, and improved mouse hearing function in an acute cisplatin-induced hearing loss model. Our findings highlight the role of NCOA4-mediated ferritinophagy in the pathogenesis of cisplatin-induced ototoxicity and provide evidence for nuciferine as a promising protective agent for treating cisplatin-induced hearing loss.

## 1. Introduction

Hearing loss is one of the most prevalent human disorders that affects communication and impacts quality of life. According to the World Health Organization (WHO), it is projected that nearly 2.5 billion people will experience some level of hearing impairment by 2050, with at least 700 million individuals requiring hearing rehabilitation [1]. Cisplatin, a highly potent antineoplastic drug, is widely used to treat a wide range of malignancies such as ovarian, bladder, lung, and head and neck solid tumors. Unfortunately, its usage is severely limited due to inevitable toxic side effects, notably nephrotoxicity, neurotoxicity, and ototoxicity [2]. More than 50% of patients developed sensorineural hearing loss after receiving cisplatin chemotherapy [3,4,5]. By targeting cochlear hair cells (HCs), spiral ganglion neurons, and the stria vascularis [6,7], cisplatin can cause permanent and irreversible hearing loss. The ototoxic effects of cisplatin are associated with the accumulation of reactive oxygen species (ROS) [8,9,10,11,12,13], DNA damage, mitochondrial dysfunction [12,14,15], and the dysregulation of inflammatory responses [16,17,18]. However, the exact mechanisms are still not very clear. To date, there are few clinically approved therapies for preventing cisplatin-induced ototoxicity. Several interventions that have demonstrated strong otoprotective effects in animal experiments, including sodium thiosulfate [19], dexamethasone [20], N-Acetylcysteine [21], amifostine [22], statins [2], multivitamins [23], and D-methionine [24], have been enrolled in clinical trials. So far, only sodium thiosulfate has been approved by the Food and Drug Administration (FDA) to mitigate cisplatin-induced hearing loss in children. However, its application is limited due to its interference with the anti-neoplastic effect of cisplatin and gastrointestinal side effects [19,25,26]. Therefore, there is an urgent need to identify novel therapeutic interventions and investigate the underlying molecular mechanisms responsible for cisplatin-induced hearing loss.

Ferroptosis, a regulated form of cell death, is characterized by the iron-dependent accumulation of lipid peroxides [27,28]. Several small molecules, such as erastin and RSL3, have been identified as triggers of ferroptosis by inhibiting the cell surface cystine/glutamate antiporter (system xc-) and glutathione peroxidase 4 (GPX4), respectively [29,30]. In conditions such as stroke [31,32,33], acute kidney injury [34,35,36], and certain neurodegenerative diseases [37,38,39], ferroptosis is considered a critical factor contributing to disease initiation or progression. The overload of redox-active iron (ferrous iron, Fe^2+^) and accumulation of lipid peroxidation products, two hallmarks of ferroptosis, result in mitochondrial dysfunction, DNA damage, impairment of membrane integrity, and, ultimately, cell death [40]. As an indispensable element of living organisms, iron has an essential role in regulating various physiological and pathological processes. Thus, iron metabolism, including iron absorption, transportation, storage, and utilization must be finely regulated to maintain normal physiological functions [41]. Ferritinophagy, a selective form of autophagy that regulates iron metabolism, refers to selective autophagic degradation of ferritin. This process is mediated by nuclear receptor coactivator 4 (NCOA4), which specifically recognizes and binds to ferritin heavy chain 1 (FTH1), a component of ferritin, and delivers it to autophagosomes for lysosomal degradation, leading to ferrous iron release [40,42,43,44]. Over-activation of ferritinophagy can result in excessive ferrous iron accumulation within cells, which may contribute to ferroptosis in two ways. Firstly, the overload of ferrous iron may provoke an intensive lipid ROS generation driven by an inorganic chemical reaction, termed the “Fenton reaction” [28,45]. Secondly, the sharply rising levels of ferrous iron elicit the over-activation of iron-containing enzymes such as lipoxygenases and acyl-CoA synthetase long-chain family member 4 (ACSL4), which further promote the lipid peroxidation [28,40]. It is reported that the pharmacological inhibition of ferroptosis using ferrostatin-1 alleviated cisplatin-induced damage in cochlear hair cells [46,47]. However, whether ferritinophagy is involved in cisplatin-induced ototoxicity still remains elusive.

Nuciferine, an active compound extracted from lotus leaf, has been shown to have various bioactivities, including anti-obesity, anti-inflammation, and anti-cancer effects [48,49,50,51]. Recent studies have demonstrated that nuciferine mitigates ferroptosis in folic acid-induced kidney injury by inhibiting iron accumulation and inflammatory cell infiltration [52], suggesting its potential as an anti-ferroptosis and anti-nephrotoxicity agent. We speculated that nuciferine might exert an anti-ferroptosis effect on cochlear hair cells. To date, there has been no investigation into the potential protective effect of nuciferine on cochlear hair cells and hearing function. In this study, we aimed to explore the anti-ferroptosis effect of nuciferine in cochlear hair cells, validate its therapeutic potential in cisplatin-induced hearing loss, and elucidate the underlying molecular mechanisms involved.

## 2. Materials and Methods

### 2.1. Animals

Five-week-old and postnatal three-day (P3) C57/BL6 wild-type mice were bought from JSJ Experimental Animal Company Ltd. After temporary rest (at least two days) in clean, quiet, and constant-temperature (22–24 °C) rooms, the adult animals were used for subsequent experiments. The initial sample size was 10 or 12 for one group, considering the “3Rs” guidelines, statistical analysis, and the possibility of animal death. Animals of both sexes were randomly divided into the control and experimental groups. Researchers were blinded to the group allocation during the experiment stages and data analysis. Atoh1-EGFP mice, in which the cochlear hair cells were labeled by EGFP fluorescence at the postnatal stage [53], were bred under specific pathogen-free conditions. We strictly adhered to ARRIVE and “3Rs” guidelines throughout all experiments [54]. Every effort was made to minimize the number of animals used. All experimental procedures were conducted according to the guidelines of the Declaration of Helsinki, and approved by the Animal Ethics Committee of Fudan University (approval code: 202107010S, date of approval: 22 July 2021).

### 2.2. Quantitative Real-Time PCR (qPCR)

All experimental procedures of qPCR were carried out adhering to the MIQE guidelines [55]. All samples used for qPCR experiments were the cochlear sensory epithelia from P3 C57/BL6 wild-type mice treated with or without cisplatin and nuciferine. Each group had 8 cochleae from different mice. All samples were fresh and had not been fixed or frozen in any way. All cochlear explants were rinsed three times with phosphate buffered saline (PBS) to ensure no drug residues. Subsequently, the cochlear explants were carefully transferred to RNase-free tubes prefilled with Trizol (Invitrogen, Carlsbad, CA, USA, 15596026CN). Then, RNA was extracted according to the manufacturer’s instructions and dissolved in the RNase-free water. During the process of RNA extraction, all efforts were made to avoid the degradation of RNA. The concentration and purity of RNA were detected via a NanoDrop2000 spectrophotometer (Thermo Scientific, Waltham, MA, USA). RNA integrity was assessed by gel electrophoresis. Then, 2 μg of RNA was reverse transcribed into cDNA using PrimeScript™ RT reagent Kit with gDNA Eraser (Takara, Kyoto, Japan, RR047A) according to the manufacturer’s instructions. qPCR was performed on a CFX-96 Real-Time PCR system (Bio-Rad, Hercules, CA, USA) using the TB Green™ PrimeScript™ RT-PCR Kit (Takara, Kyoto, Japan, RR820A). The protocols for RNA extractions, cDNA reverse transcription, and qPCR experiments are provided in the Supplementary Materials. All primers used in this study are listed in Table 1. The specificity of the primers was determined by observing the melting curve. Relative gene expression compared to *Gapdh* was quantified using the 2^−ΔΔ^Cq method.

### 2.3. HEI-OC1 Cells Culture and Cisplatin Treatments

HEI-OC1 cells were incubated in DMEM/F12 medium (Gibco, Logan, UT, USA) with 5% fetal bovine serum (FBS, Gibco, Logan, UT, USA) and 100 μg/mL ampicillin at 33 °C with 10% CO_2_. HEI-OC1 cells were treated with 30 μM cisplatin (Sigma-Aldrich, St. Louis, MO, USA, Cat.no. 15663-27-1) for 24 h with or without nuciferine (Selleckchem, Houston, TX, USA, s3821). The dosage of cisplatin used in this study was determined based on our previous work, which demonstrated that 30 μM cisplatin induced the maximum damage intensity in HEI-OC1 cells [56].

### 2.4. The Preparation Protocol of Nuciferine Solution

According to the instructions from Selleck Chemicals (Houston, TX, USA), nuciferine can only be dissolved in dimethyl sulfoxide (DMSO), not in water or ethanol. In the in vitro experiments, nuciferine was dissolved in DMSO at a stock concentration of 20 mM and further diluted in the cell culture medium to a desired concentration. In the in vivo experiments, nuciferine was dissolved in DMSO at a stock concentration of 37 mM by vortex oscillation and then diluted in solvent (10% DMSO + 90% corn oil), and finally injected into mice intraperitoneally. 

### 2.5. Cochlear Explant Culture

The cochlear sensory epithelia from P3 C57/BL6 wild-type mice were carefully dissected and collected in PBS. Then the dissected cochlear explants were transferred to a Cell-Tak (BD Bioscience, Franklin Lakes, NJ, USA, 354240)-coated slide, and placed on ice for 5–10 min to ensure tight adhesion. Finally, the well-adhered cochlear explants were cultured in DMEM/F12 medium supplemented with N2 (Life Technologies, Carlsbad, CA, USA), B27 (Life Technologies, Carlsbad, CA, USA), and 100 μg/mL ampicillin overnight at 37 °C.

### 2.6. RSL3 Experiments

The cochlear sensory epithelia from P3 C57/BL6 wild-type mice were dissected and cultured as described above. After 3~4 h of incubation in medium for stability, the cochlear sensory epithelia were treated with 5 μM RSL3 (Sigma-Aldrich, St. Louis, MO, USA, Cat.no. 1219810-16-8) for 6 h with or without co-treatment with nuciferine (60 μM or 90 μM). Then the explants of each group were harvested for hair cell analysis after 24 h recovery. 

### 2.7. Detection of Iron in Hair Cells

To detect intracellular Fe^2+^, FerroOrange (Dojindo, Kumamoto, Japan, F374) was used according to the manufacturer’s protocol. P3 cochleae from Atoh1-EGFP mice were stained with FerroOrange at a final concentration of 1 μM for 30 min at 37 °C after cisplatin treatment (30 μM, 18 h) with or without nuciferine. The presence of intracellular Fe^2+^ was observed under a Leica SP8 confocal laser scanning microscope (Leica Microsystems, Wetzlar, Germany). The fluorescence intensity was measured and analyzed using ImageJ (National Institutes of Health).

### 2.8. Immunofluorescence

The HEI-OC1 cells and cochlear explants samples were fixed in 4% paraformaldehyde at room temperature for 30 min. Cochlear samples from adult mice were fixed in 4% paraformaldehyde at room temperature for 2 h. After complete fixation, samples were rinsed three times with PBS, then permeabilized and blocked with PBST (PBS with 1% Triton X-100) containing 10% donkey serum for 1 h. Well-prepared samples were immersed in PBST with 1% donkey serum and certain primary antibodies overnight at 4 °C. The primary antibodies used in this study included anti-myosin VIIA (Myo7a) antibodies (Proteus Biosciences, Ramona, CA, USA, 25-6790, AB_10015251, 1:1000 dilution), anti-NCOA4 antibodies (Invitrogen, Carlsbad, CA, USA, PA5-96398, AB_2808200, 1:50 dilution), anti-FTH antibodies (Cell Signaling, Danvers, MA, USA, 3998, AB_1903974, 1:100 dilution), anti-LAMP1 antibodies (Santa Cruz, Dallas, TX, USA, sc-20011, AB_626853, 1:50 dilution), and anti-C-Terminal Binding Protein (CtBP2, BD Biosciences, Franklin Lakes, NJ, USA, 612044, AB_399431, 1:1000 dilution). Specimens were then rinsed three times with PBS and incubated with the corresponding fluorescent secondary antibody for 12 h at 4 °C in the dark. DAPI (Sigma-Aldrich, St. Louis, MO, USA, D9542, 1:800 dilution) was used to label nuclei. All samples were observed and documented using confocal fluorescence microscopy (Leica Microsystems, SP8).

### 2.9. Small Interfering RNA (siRNA) and Transfection

Predesigned siRNAs against mouse *Ncoa4* (Gene ID:27057), and negative control siRNA (scrambled siRNA, siScramble) were purchased from GenePharma. The sense strands of siRNAs against *Ncoa4* were as follows: Duplex 1 Sense Strand: 5′-GGAGUAUACUCAGAACAAATT-3′, Duplex 2 Sense Strand: 5′-CCACGUCAUAAAGAAUAGUTT-3′, and Duplex 3 Sense Strand: 5′-CCCUACAAGAAGAACGUAATT-3′. Cochlear explants were transiently transfected with 100 nM of siRNA construct in the Opti-MEM medium with the presence of Lipofectamine 3000 Reagents (Thermo Fisher Scientific, Waltham, MA, USA) according to the manufacturer’s protocol. After incubation at 37 °C and 5% CO_2_ for 48 h, the cochlear explants were further treated with cisplatin for 24 h. The knockdown of *Ncoa4* expression was confirmed by qPCR analysis.

### 2.10. In Vivo Acute Cisplatin Challenge and Drug Treatment

For validation of the otoprotective effect of nuciferine in cisplatin-treated adult mice, we established an acute cisplatin challenge model as previously described [56]. After baseline hearing evaluation, 30 mg/kg cisplatin was administered intraperitoneally. A quantity of 15 mg/kg nuciferine was given intraperitoneally 2 h before and 24 h after cisplatin injection [48,52,57]. Warm saline was administered to mitigate cisplatin-induced dehydration and renal toxicity as described previously [58]. Hearing thresholds were measured 3 days after cisplatin treatment [56,59].

### 2.11. Auditory Brainstem Response (ABR)

ABR was used to assess mouse auditory function using the TDT’s RZ6 workstation [60,61,62] (Tucker Davis Technology, Alachua, FL, USA). Hearing thresholds of 8, 16, 24, and 32 kHz were evaluated using open-field loudspeakers. In brief, ABRs were recorded via three subdermal needle electrodes placed on the vertex of the skull (active electrode), the mastoid area of the right ear (reference electrode), and the back (ground electrode). The hearing thresholds, the amplitude, and latency of ABR wave I were measured and determined using Bio-Sig RP software (Version 4.4.10, Tucker Davis Technology, Alachua, FL, USA) as previously described [56].

### 2.12. Image Processing

All samples were observed and documented using confocal fluorescence microscopy (Leica Microsystems, SP8). The pixel size of each original image derived from confocal fluorescence microscopy was 1024 × 1024. All final images in figures were processed by Image J, and cropped by Adobe Photoshop in the ratio of 4:3 or 3:2. During the image acquisition using confocal fluorescence microscopy (Leica Microsystems, SP8), Z stack was utilized throughout the process. According to the respective characteristics of the zones of interest in the cochlea, we chose different scanning thicknesses per Z stack layer: 1 μm for hair cells; 0.3 μm for lysosomes in HEI-OC1 cells; and 0.5 μm for Ctbp2 positive puncta. Additionally, a 40× oil lens was used for hair cell observation and a 63× oil lens with 3× zoom was used for lysosomes and Ctbp2 positive puncta imaging. In all groups of the same experiment, the thresholding of fluorescence intensity was kept at the same level to ensure the comparability of results. The length of ROI (Region of Interest) lines in FerroOrange and lysosomes images were 65 μm and 15 μm, respectively. All ROI lines were randomly selected.

### 2.13. Cell Counting

**Figure 1:**
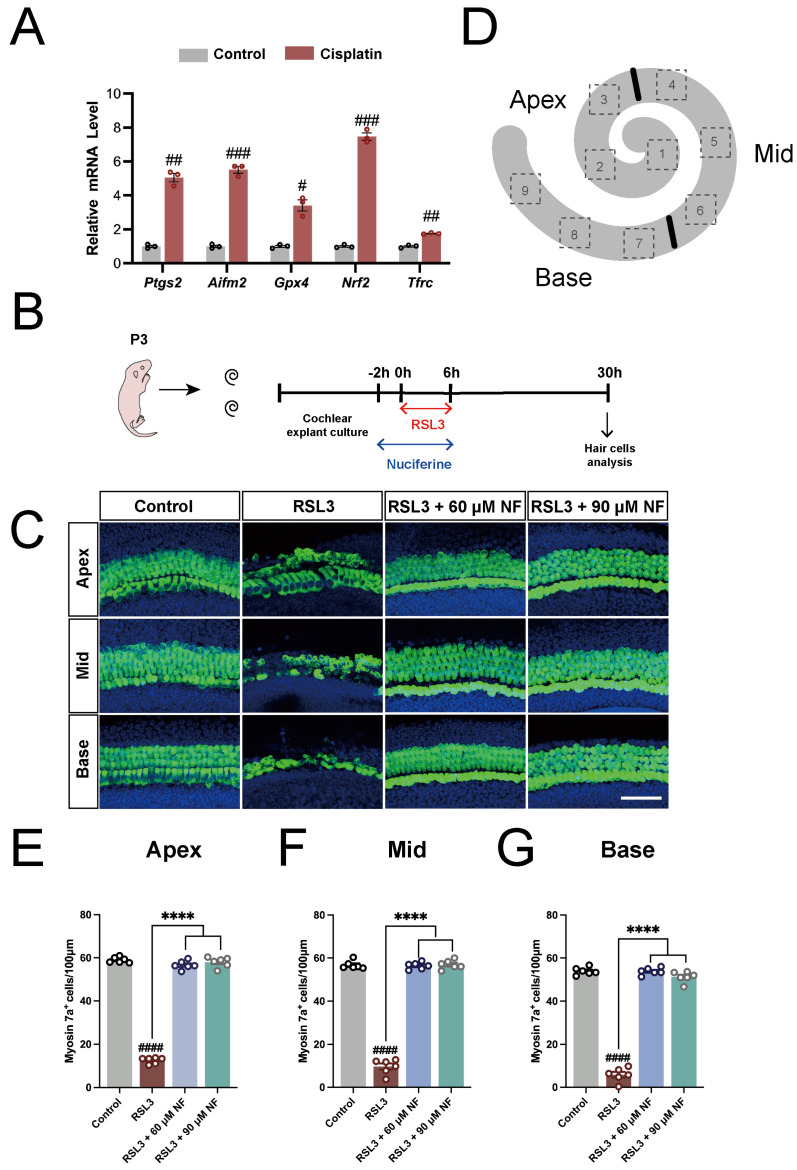
Nuciferine potently suppressed RSL3-induced ferroptosis of cochlear hair cells. (**A**) Quantitative real-time PCR results showed the relative mRNA levels of *Ptgs2*, *Aifm2*/*Fsp1*, *Gpx4*, *Nrf2*, and *Tfrc* in hair cells after 24 h cisplatin treatment. *n* = 3. (**B**) Diagram of the assay for (**C**–**G**). (**C**) Immunofluorescence staining with Myosin7a (green) and DAPI (blue) in the apical, middle, and basal turns of the cochleae from control, RSL3-only, RSL3 with 60 μM nuciferine co-treatment, and RSL3 with 90 μM nuciferine co-treatment groups. (**D**) The scheme for cell counting. We counted cells in 9 serial counting boxes (100 μm length, 3 boxes in the apex (number 1–3), 3 boxes in the mid (number 4–6), and 3 boxes in the base (number 7–9)) in each cochlear explant. (**E**–**G**) Quantification of Myosin7a+ hair cells in different groups. *n* = 6. The data are shown as mean ± SEM. # indicates *p* < 0.05, ## indicates *p* < 0.01, ### indicates *p* < 0.001, #### and **** indicates *p* < 0.0001, ns indicates not significant. Octothorpe (#) indicates statistical difference vs. the control group. Stars (*) indicate statistical difference vs. the RSL3 group. Bar = 50 μm. NF, nuciferine.

Immunofluorescence staining-positive cells were counted as previously described by selecting nine separate segments from the apical to the basal turn along the entire cochlea [56,60]. The details were as follows: the Myosin7a positive cells (HCs) were counted as shown in Figure 1D. We counted cells in nine serial counting boxes (100 μm length, 3 boxes in the apex, 3 boxes in the mid, and 3 boxes in the base) in each cochlear explant. The cell numbers of 3 counting boxes (apex, mid, or base) in each cochlear explant were averaged, then the average of six cochlear explants was further analyzed using GraphPad Prism 9 software to obtain the mean and SEM of the cell number in each group, and the statistical graphs.

### 2.14. Statistical Analysis

The Student’s *t*-test was used to analyze differences between two groups, and one-way ANOVA followed by the Bonferroni post-test was used for comparisons among three or more groups. Two-way ANOVA followed by the Bonferroni post-test was used for comparisons of ABR hearing threshold, threshold shift, and latency of ABR wave I. The rank sum test (Mann–Whitney U test) was used to analyze data that did not conform to a normal distribution. Data are presented as the mean ± SEM. A *p*-value < 0.05 was considered significant (* and # indicate *p* < 0.05, ** and ## indicate *p* < 0.01, *** and ### indicate *p* < 0.001, and **** and #### indicate *p* < 0.0001).

## 3. Results

### 3.1. Nuciferine Inhibited RSL3-Induced Hair Cell Ferroptosis

Previous studies have noted that cisplatin induces an overload of ferrous iron and accumulation of lipid reactive oxygen species in HEI-OC1 cells [46], a HC-like cell line [63,64,65]. Ferrostatin-1, a ferroptosis inhibitor, can protect HEI-OC1 cells and cochlear hair cells against cisplatin-induced cell death. To confirm whether cisplatin can induce ferroptosis in cochlear HCs, cochlear explants were treated with cisplatin for 24 h. Subsequently, the relative mRNA levels of representative ferroptosis-related genes were assessed via qPCR. The data showed that the expression levels of the ferroptosis marker *Ptgs2*, ferroptosis suppressor protein 1 (*Fsp1*, also known as *Aifm2*), glutathione peroxidase 4 (*Gpx4*), nuclear factor erythroid 2-related factor 2 (*Nrf2*), and transferrin receptor protein 1(*Tfr1*, also known as *Tfrc*) were all significantly upregulated after cisplatin treatment (Figure 1A), confirming that ferroptosis was involved in cisplatin-induced ototoxicity.

Next, we investigated whether nuciferine exhibits an anti-ferroptosis effect in cochlear HCs using an RSL3-induced HC injury model. As shown in Figure 1B, cochlear explants from P3 C57BL/6 mice were treated with 5 μM RSL3, a well-known ferroptosis inducer, for 6 h, with or without nuciferine co-treatment (60 μM or 90 μM). After the washout of nuciferine and RSL3, explants were further incubated for another 24 h before harvest. Myosin7a was used as a marker of cochlear HCs. The treatment of RSL3 resulted in massive hair cell loss (Figure 1C–G). Notably, co-treatment with nuciferine significantly ameliorated RSL3-induced hair cell damage, as indicated by the improved quantity, morphology, and arrangement of HCs (Figure 1C–G), which suggested that nuciferine protected cochlear HCs against RSL3-induced ferroptosis.

### 3.2. Nuciferine Protected Cochlear HCs against Cisplatin-Induced Ototoxicity In Vitro

**Figure 2:**
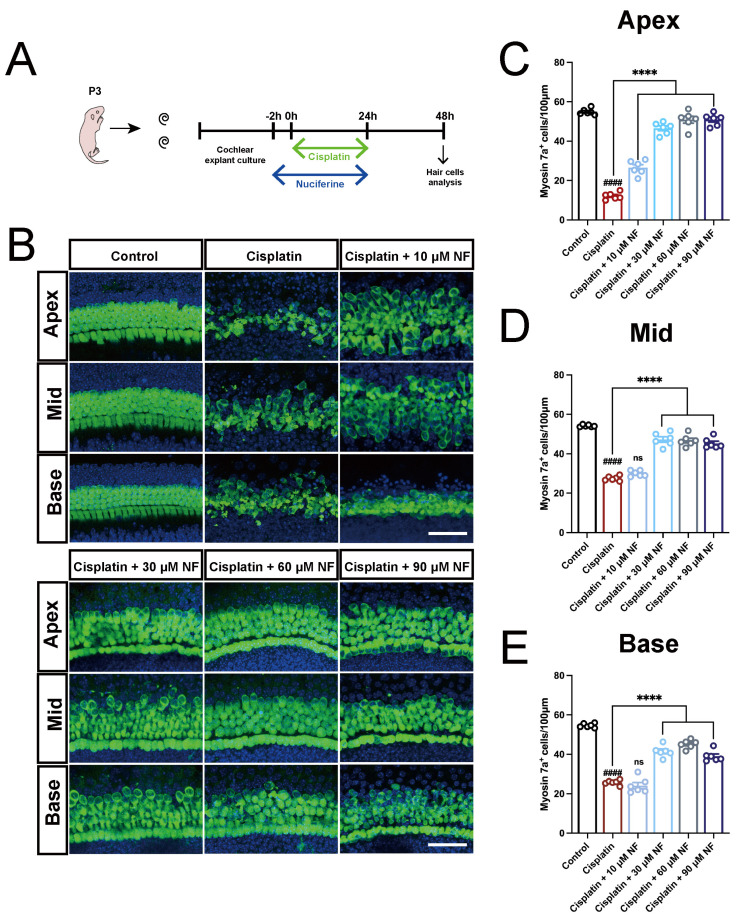
Nuciferine protected against cisplatin-induced ototoxicity in vitro. (**A**) Diagram of the assay for (**B**–**E**). (**B**) Immunofluorescence staining with Myosin7a (green) and DAPI (blue) in the apical, middle, and basal turns of the cochleae from six groups: control, cisplatin (30 μM cisplatin only), and cisplatin co-treated with different concentrations of nuciferine (10 μM, 30 μM, 60 μM, and 90 μM, respectively). (**C**–**E**) Quantification of Myosin7a+ hair cells in different groups. *n* = 6 cochleae from independent mice. The data are shown as mean ± SEM. #### and **** indicate *p* < 0.0001, ns indicates not significant. Octothorpe (#) indicates statistical difference vs. the control group. Stars (*) indicate statistical difference vs. the cisplatin group. Bar = 50 μm. NF, nuciferine.

Based on the potent inhibitory effect of nuciferine on hair cell ferroptosis, we explored whether nuciferine could rescue the hair cell damage caused by cisplatin. Cochlear explants were treated with 30 μM cisplatin for 24 h, with or without co-treatment with nuciferine at a series of concentrations (10, 30, 60, and 90 μM) (Figure 2A). As shown in Figure 2B–E, cisplatin treatment resulted in tremendous cell loss, morphological changes, and disarrangement of cochlear hair cells. However, significantly more hair cells were preserved in nuciferine co-treatment groups, at concentrations of 30, 60, and 90 μM. The morphology and arrangement of hair cells in the nuciferine co-treatment groups were much better than those in the cisplatin-alone group. The protective effect of 60 μM nuciferine on hair cells was stronger than that of 90 μM nuciferine, demonstrated by a more numerous and better-organized residual hair cells. We concluded that 60 μM nuciferine is more capable of normalizing the morphology and reducing the loss of hair cells. These results indicated that nuciferine had a potent protective effect against cisplatin-induced hair cell damage and demonstrated that nuciferine was a promising otoprotectant for cisplatin-induced ototoxicity.

**Figure 3:**
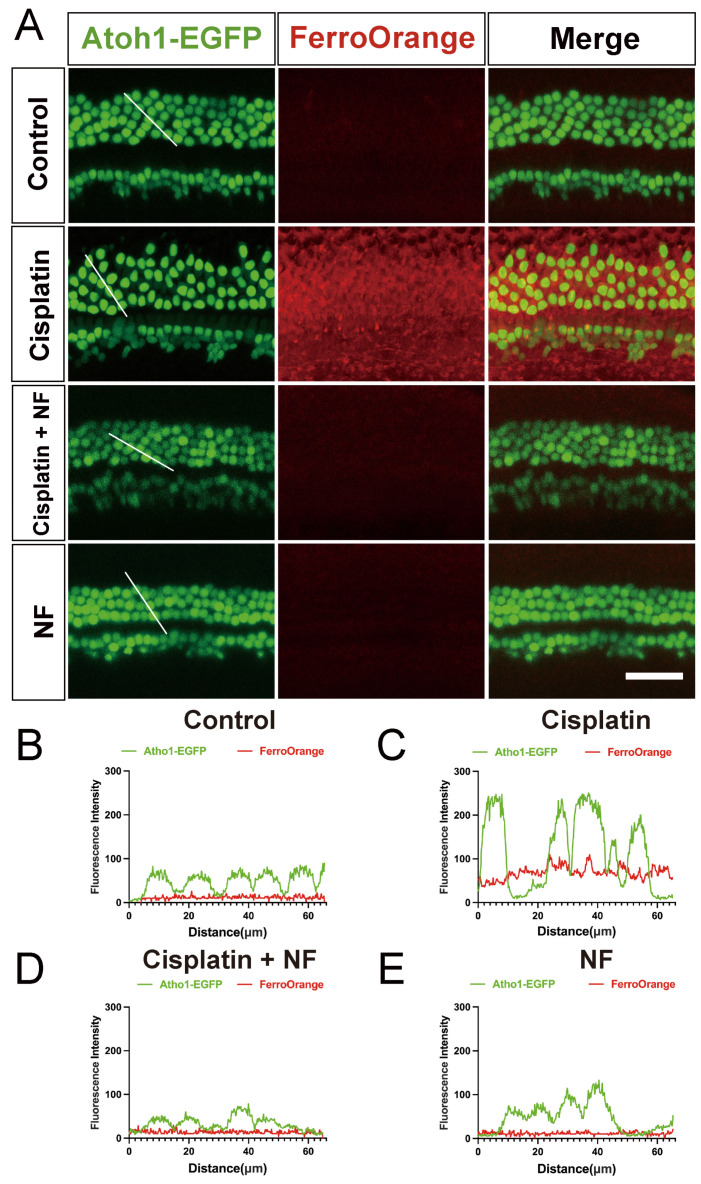
Nuciferine alleviated iron accumulation in hair cells in vitro. (**A**) Representative images of Atoh1-EGFP (green), FerroOrange (red) fluorescence in the middle turn of the cochleae. FerroOrange was used to assess the Fe^2+^ levels in hair cells. Atoh1-EGFP-positive cells were cochlear hair cells. The samples were divided into four groups: control, cisplatin (30 μM cisplatin only), cisplatin co-treated with 60 μM nuciferine, and 60 μM nuciferine only. (**B**–**E**) The fluorescence intensity of Atho1-EGFP and FerroOrange along the white line in (**A**): control group, cisplatin-alone group, cisplatin with nuciferine co-treatment group, and nuciferine-alone group. The fluorescence intensity was quantified using ImageJ software (Version 2.9.0/1.54f). *n* = 6 cochleae from independent mice. The length of white line is 65 μm. Bar = 50 μm. NF, nuciferine.

Given that iron accumulation is one of the key characteristics of ferroptosis, we detected the level of intracellular labile Fe^2+^ in cisplatin-damaged hair cells using FerroOrange probes. Cochlear explants from P3 Atoh1-EGFP mice were used, so that cochlear hair cells could be clearly seen under the fluorescence microscope. As expected, cisplatin treatment induced an excessive increase in labile Fe^2+^ levels in cochlear hair cells (Figure 3A–E). Conversely, co-treatment of nuciferine significantly suppressed the aberrant increase in the Fe^2+^ level induced by cisplatin (Figure 3A–E), indicating that nuciferine inhibited the overload of labile Fe^2+^ in hair cells caused by cisplatin.

### 3.3. Nuciferine Inhibited Cisplatin-Induced Ferritinophagy in Cochlear Hair Cells

**Figure 4:**
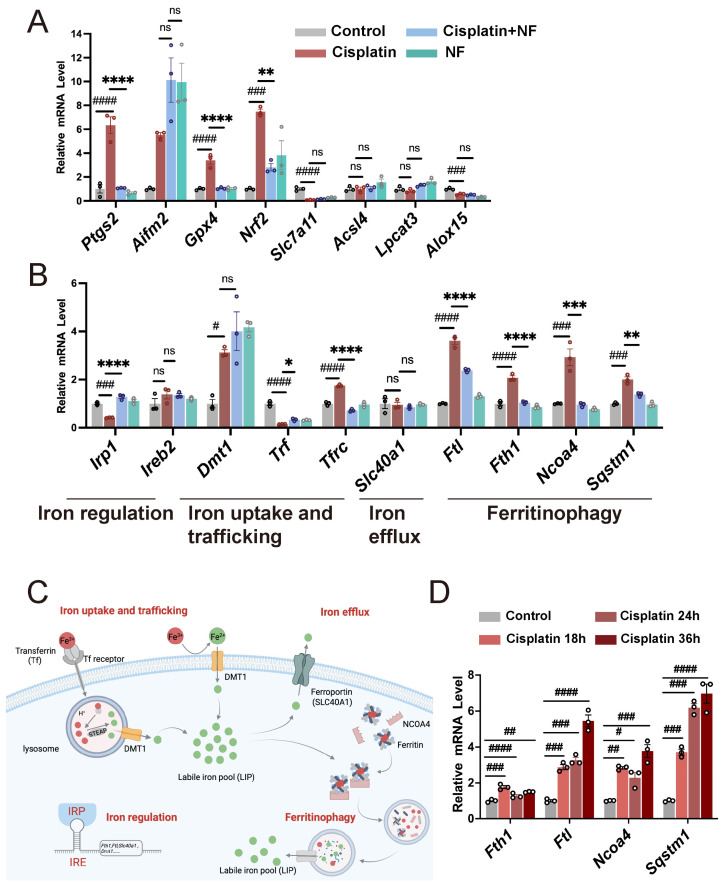
Nuciferine inhibited NCOA4-mediated ferritinophagy in cochlear hair cells. (**A**,**B**) Relative mRNA levels of ferroptosis-related genes in cochlear explants with cisplatin and nuciferine treatment for 24 h. (**C**) The iron metabolism schematic diagram. (**D**) Quantitative real-time PCR analysis showed the relative mRNA levels of ferritinophagy-related genes in cochlear explants treated with cisplatin for 0 h (control), 18 h, 24 h, and 36 h, respectively. *n* = 3. The data are shown as mean ± SEM. # and * indicate *p* < 0.05, ## and ** indicate *p* < 0.01, ### and *** indicate *p* < 0.001, #### and **** indicate *p* < 0.0001, ns indicates not significant. Octothorpe (#) indicates statistical difference vs. the control group. Stars (*) indicate statistical difference vs. the cisplatin group. NF, nuciferine.

We next sought to understand the underlying mechanisms of how nuciferine inhibited cisplatin-induced hair cell ferroptosis. We first examined the expression changes in representative ferroptosis-related genes, including antioxidant genes like *Gpx4* and *Nrf2*, lipid peroxidation-promoting genes like *Acsl4* and *Lpcat3*, and iron homeostasis regulatory genes like *Slc40a1* and *Ncoa4* (Figure 4A,B). Among these genes, ferritinophagy regulatory genes, including *Ftl*, *Fth1*, *Ncoa4*, and *Sqstm1*, were the genes most affected by cisplatin and nuciferine treatment. As shown in Figure 4B, the mRNA levels of ferritin heavy chain (FTH) and ferritin light chain (FTL), which collectively form a 24-subunit macromolecular iron-storage complex (ferritin) [66], were both significantly upregulated upon cisplatin treatment and downregulated by nuciferine co-treatment. Moreover, the mRNA levels of NCOA4, which acts as the selective cargo receptor of ferritinophagy by specifically binding to FTH1 and delivering ferritin to the lysosome for degradation (Figure 4C) [67,68], remarkably increased after cisplatin injury and were rescued by nuciferine co-treatment. Furthermore, the mRNA expression of the autophagy-related protein P62 (encoded by *Sqstm1*) [69] changed in the same way (Figure 4B). The above results raised the possibility that ferritinophagy might be involved in the cisplatin-induced hair cell damage and nuciferine may protect against cisplatin-induced ototoxicity by suppressing this process.

To verify whether cisplatin triggered ferritinophagy in HCs, we measured the expression changes in *Ftl*, *Fth1*, *Ncoa4*, and *Sqstm1* at a series of time points (18, 24, and 36 h) after the beginning of cisplatin exposure via qPCR. As shown in Figure 4D, the mRNA levels of these genes exhibited an increasing tendency in a time-dependent manner, further confirming that the process of ferritinophagy was activated by cisplatin injury.

**Figure 5:**
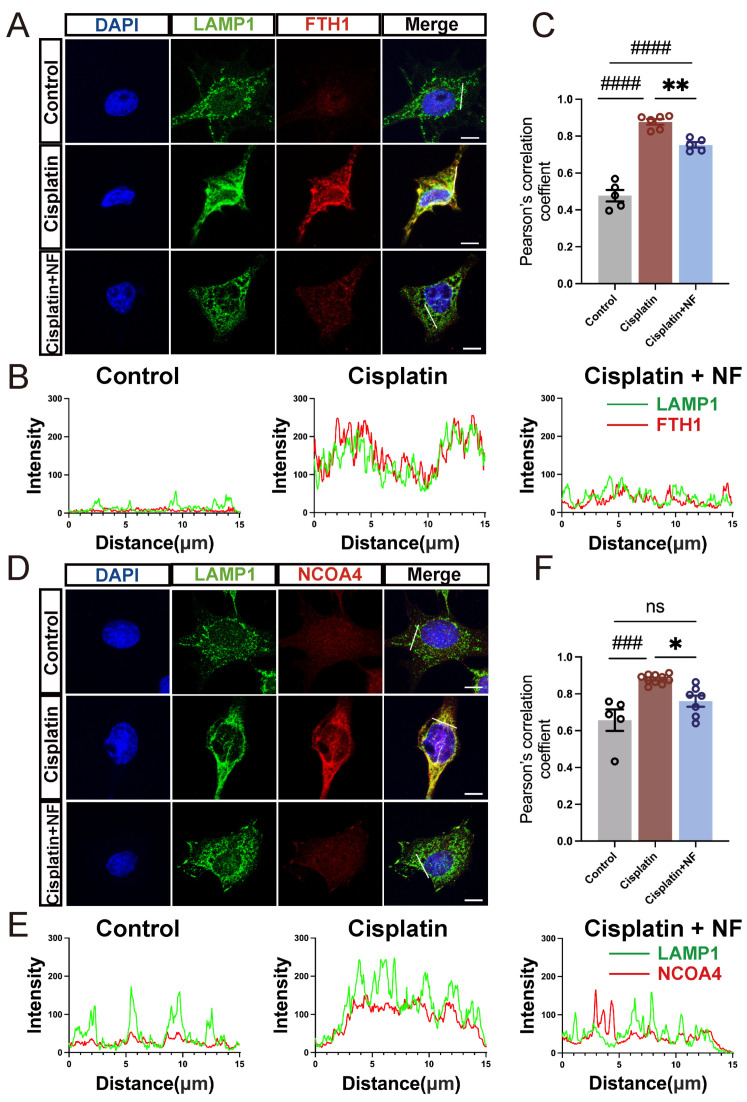
Nuciferine inhibited NCOA4-mediated ferritinophagy in HEI-OC1 cells. (**A**) Immunofluorescence staining with LAMP1 (green), FTH1 (red), and DAPI (blue) in the HEI-OC1 cells. (**B**) Quantitative immunofluorescence intensity along the white line in (**A**). (**C**) Pearson’s correlation coefficients quantified the extent of colocalization of LAMP1 and FTH1 in respective groups in (**A**) (*n* ≥ 5 cells for each group). (**D**) Immunofluorescence staining with LAMP1 (green), NCOA4 (red), and DAPI (bule) in the HEI-OC1 cells. (**E**) Quantitative immunofluorescence intensity along the white line in (**D**). (**F**) Pearson’s correlation coefficients quantified the extent of colocalization of LAMP1 and NCOA4 in respective groups in (**D**) (*n* ≥ 5 cells for each group). The data are shown as mean ± SEM. * indicates *p* < 0.05, ** indicates *p* < 0.01, ### indicates *p* < 0.001, #### indicates *p* < 0.0001, ns indicates not significant. Octothorpe (#) indicates statistical difference vs. the control group. Stars (*) indicate statistical difference vs. the cisplatin group. The length of white line is 15 μm. Bar = 10 μm. NF, nuciferine.

We further used HEI-OC1 cells, the HC-like cell line, to explore the subcellular process of ferritinophagy upon cisplatin damage. Dual immunofluorescence labeling of FTH1/NCOA4 and lysosome-associated membrane protein 1 (LAMP1, a marker of lysosomes) was performed to observe the localization of FTH1 and NCOA4 in the lysosomes. In response to cisplatin injury, the co-localization of FTH1 with LAMP1 significantly increased (*p* < 0.0001, Figure 5A–C), indicating that more ferritins were delivered to lysosomes for degradation upon cisplatin exposure. Nuciferine co-treatment significantly reduced the co-localization of FTH1 with LAMP1 caused by cisplatin injury (*p* < 0.01, Figure 5A–C). Consistent with the above data, cisplatin injury enhanced the co-localization of NCOA4 and LAMP1, which was remarkably alleviated by nuciferine co-treatment (Figure 5D–F). These data suggested that nuciferine inhibited cisplatin-induced over-activation of NCOA4-mediated ferritinophagy.

### 3.4. Knocking Down of Ncoa4 Alleviated Cisplatin-Induced Ototoxicity In Vitro

**Figure 6:**
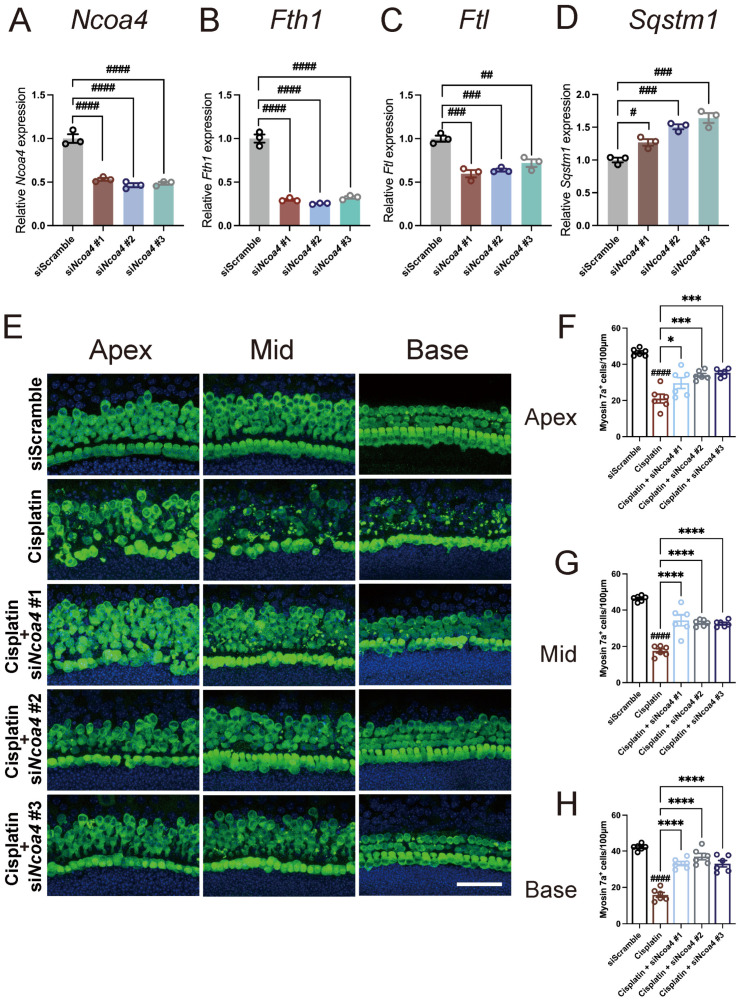
Genetic knockdown of *Ncoa4* alleviated cisplatin-induced ototoxicity. (**A**–**D**) The relative mRNA levels of ferritinophagy-related genes were detected by quantitative real-time PCR in the negative control (siScramble) and *Ncoa4* knockdown (si*Ncoa4*) cochlear explants. (**E**) Representative immunofluorescence images with Myosin7a (green) and DAPI (blue) in the apical, middle, and basal turns of the cochleae after co-treatment with the *Ncoa4* siRNA and cisplatin. (**F**–**H**) Quantification of Myosin7a+ hair cells in different groups. *n* = 6 cochleae from independent mice. The data are shown as mean ± SEM. # and * indicate *p* < 0.05, ## indicates *p* < 0.01, ### and *** indicate *p* < 0.001, #### and **** indicate *p* < 0.0001. Octothorpe (#) indicates statistical difference vs. the siScramble group. Stars (*) indicate statistical difference vs. the cisplatin group. Bar = 50 μm.

Ferritinophagy is a highly orchestrated regulatory process critical for intracellular iron bioavailability, depending on the precise recognition and direct interaction between a conserved C-terminal domain of NCOA4 and a key conserved residue on FTH1 [68]. Continuous transference of ferritin to lysosomes for degradation results in a significant release of free ferrous iron, thus promoting the generation and accumulation of free radicals and lipid peroxides via Fenton reactions [67,70,71]. Although accumulating evidence has shown that ferritinophagy plays a critical role in the pathological mechanisms of ischemic stroke, Parkinson’s disease, and other diseases [72,73,74,75], whether ferritinophagy is involved in cisplatin-induced ototoxicity is still unknown. The above data indicated that nuciferine inhibited ferritinophagy-related genes expression and ferritin degradation, but whether direct inhibition of ferritinophagy can generate a protective effect against cisplatin-induced ototoxicity is unclear. To answer this question, the expression of NCOA4 was knocked down using siRNA in cochlear explants to suppress ferritinophagy. As qPCR data (Figure 6A–D) showed, in si*Ncoa4* treatment groups, the gene expressions of *Ncoa4*, *Fth1*, and *Ftl* were all significantly reduced, and the mRNA levels of autophagy receptor *Sqstm1* were correspondingly upregulated, indicating that knockdown of *Ncoa4* effectively suppressed the process of ferritinophagy in hair cells. As a result, the hair cell damage induced by cisplatin was notably attenuated by *Ncoa4* knockdown, as demonstrated by the significantly larger number of residual Myosin7a-positive cells in the three si*Ncoa4* RNA groups after cisplatin exposure (Figure 6E–H). These results confirmed that NCOA4-mediated ferritinophagy was involved in cisplatin-induced ototoxicity and suggested that ferritinophagy inhibition is a promising therapeutic target for treating cisplatin-induced hearing loss.

### 3.5. Nuciferine Protected against Cisplatin-Induced Hearing Loss

**Figure 7:**
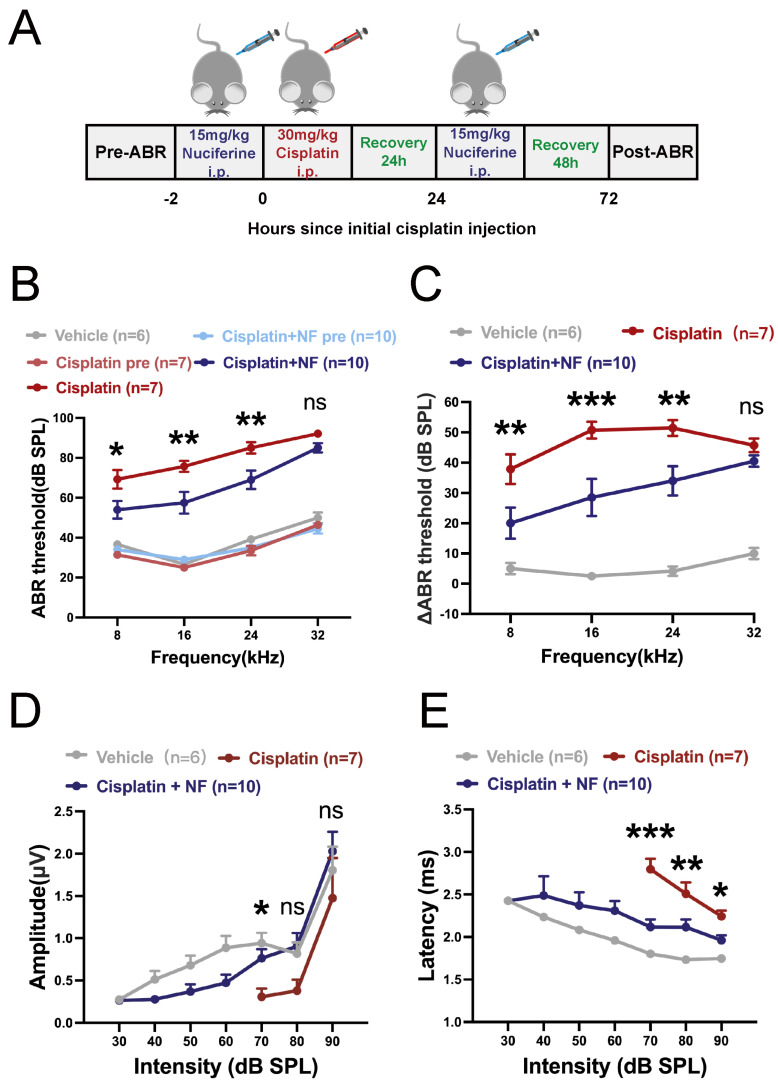
Nuciferine protected against cisplatin-induced hearing loss. (**A**) Flow chart of in vivo experiments in mice. Five-week-old mice were injected intraperitoneally with 30 mg/kg cisplatin. Nuciferine (15 mg/kg) was administered intraperitoneally 2 h before and 24 h after cisplatin administration. The same dose of solvent was administered intraperitoneally in the vehicle group and the cisplatin group. Hearing function was measured and cochleae were harvested 72 h after the first cisplatin injection. (**B**) ABR thresholds at 72 h after cisplatin treatment. The meaning of “cisplatin pre” and “cisplatin + NF pre” indicates the baseline ABR threshold before cisplatin treatment. (**C**) Statistical data of ABR threshold shifts after cisplatin injury. (**D**,**E**) Statistical data of the amplitudes and latencies of ABR wave I at 16 kHz in different groups. *n* = 6, 7, and 10 for vehicle, cisplatin, and cisplatin +NF groups, respectively. The data are shown as mean ± SEM. * indicates *p* < 0.05, ** indicates *p* < 0.01, *** indicates *p* < 0.001, ns indicates not significant. Stars (*) indicate statistical difference vs. the cisplatin group. NF, nuciferine.

Next, we explored the protective potential of nuciferine against cisplatin-induced hearing loss. An acute cisplatin injury model was established by intraperitoneally administering a dose (30 mg/kg) of cisplatin once to induce acute hearing loss in 5-week-old C57BL/6 mice [59]. Nuciferine (15 mg/kg, i.p.) was injected 2 h before and 24 h after cisplatin administration. Auditory brainstem response (ABR) tests were conducted 72 h after cisplatin injection (Figure 7A). The same amount of solvent vehicle (10% DMSO + 90% corn oil) was delivered intraperitoneally to the vehicle group and the cisplatin-only group to exclude the influence of drug delivery vehicle on hearing analysis. As shown in Figure 7B–E, the ABR thresholds significantly elevated after cisplatin injury. However, the co-treatment of nuciferine significantly reduced threshold elevations at 8, 16, and 24 kHz frequencies (*p* < 0.05, Figure 7B). Threshold shifts were significantly smaller in the nuciferine co-treatment group at 8, 16, and 24 kHz frequencies (*p* < 0.01, Figure 7C) compared to the cisplatin-only group. Analysis of ABR wave I at 16 kHz frequency revealed a significant amplitude reduction and prolonged latency after cisplatin treatment, indicating potential changes in the function of the synapses between inner hair cells (IHCs) and spiral ganglion neurons (SGNs). Nuciferine co-treatment significantly reduced the cisplatin-induced amplitude decrease in wave I at 70 dB (*p* < 0.05, Figure 7D), and attenuated the cisplatin-induced latency prolongation of ABR wave I at 70, 80, and 90 dB (*p* < 0.05, Figure 7E).

**Figure 8:**
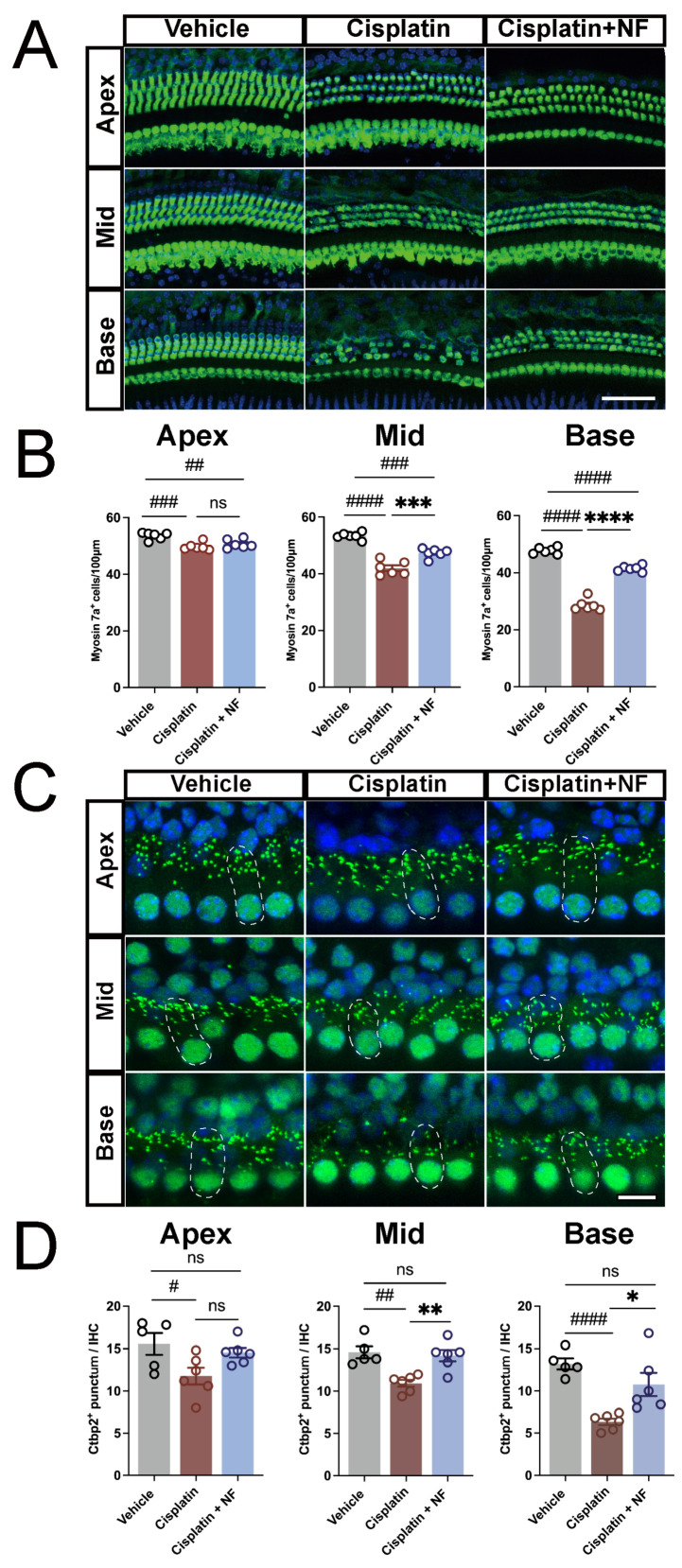
Nuciferine reduced cochlear hair cell loss and ribbon synapse damage in vivo. (**A**) Representative images of Myosin7a+ hair cells after the administration of cisplatin and nuciferine. (**B**) Quantification of Myosin7a+ hair cells in apical, middle, and basal turns. (**C**) Representative images of Ctbp2 positive puncta in the IHC-SGN synapses. The typical image of IHC and surrounding Ctbp2 positive puncta are outlined with dashed lines. (**D**) Quantification of Ctbp2 positive puncta per IHC from different groups. *n* = 6. The data are shown as mean ± SEM. # and * indicate *p* < 0.05, ## and ** indicate *p* < 0.01, ### and *** indicate *p* < 0.001, #### and **** indicate *p* < 0.0001, ns indicates not significant. Octothorpe (#) indicate statistical difference vs. the vehicle group. Stars (*) indicate statistical difference vs. the cisplatin group. Bar = 50 μm in A, and 10 μm in C. NF, nuciferine.

Morphological analysis showed that nuciferine significantly ameliorated the cisplatin-induced hair cell loss at the middle and basal turns (*p* < 0.001, Figure 8A,B). Considering the effect of nuciferine on ABR wave I amplitude and latency, we explored whether it can protect the synapses between IHCs-SGNs, another vulnerable cochlear component of cisplatin injury [76,77], by immunostaining of Ctbp2, a presynaptic marker of IHCs-SGNs synapse. As demonstrated in Figure 8C,D, after cisplatin treatment, the numbers of Ctbp2+ puncta were significantly lower than those in the vehicle group in all three turns. However, nuciferine co-treatment remarkably alleviated the cisplatin-induced loss of Ctbp2+ puncta in the middle and basal cochlear turns (*p* < 0.05, Figure 8D), suggesting that nuciferine treatment preserved more ribbon synapses from cisplatin injury. These results indicated that nuciferine could protect against hair cell degeneration and cisplatin-induced hearing loss.

## 4. Discussion

Cisplatin is a frequently used chemotherapeutic medicine for cancer treatment. Bilateral and permanent sensorineural hearing loss is recognized as one of the most serious side effects of cisplatin, but there are few FDA-approved medicines to prevent it. Therefore, finding effective drugs to ameliorate cisplatin-induced ototoxicity is urgently needed. Previous studies have reported that ferroptosis inhibitor ferrostatin-1, which can inhibit the generation of lipid peroxides [29,78], protects against cisplatin-induced ototoxicity in HEI-OC1 cells and cochlear hair cells [46]. However, the deeper understanding of the underlying mechanisms among iron homeostasis, ferroptosis, and cisplatin-induced ototoxicity remains elusive. In the current work, we confirmed that cisplatin triggered the overload of divalent iron and overwhelming ferroptosis in cochlear hair cells (Figure 1, Figure 2 and Figure 3), which is consistent with previous studies. Ferritinophagy, a selective type of autophagy mediated by the selective cargo receptor NCOA4, can induce ferroptosis by degrading ferritin and inducing iron overload [67]. Our data showed that NCOA4-mediated ferritinophagy was over-activated in HEI-OC1 cells and cochlear hair cells, demonstrated by the upregulation of ferritinophagy-related genes in cisplatin damaged hair cells and increased localization of NCOA4 and FTH1 in LAPM1+ lysosomes of HEI-OC1 cells (Figure 4 and Figure 5). Additionally, we demonstrated that knocking down *Ncoa4* enabled hair cells to be more resistant to cisplatin-induced ototoxicity, as indicated by the more residual Myosin7a positive cells upon cisplatin exposure (Figure 6). All of these findings collectively confirmed the critical role of NCOA4-mediated ferritinophagy in the pathogenesis of cisplatin-induced ototoxicity. Targeting ferritinophagy provides a promising approach for treating cisplatin-induced hearing loss.

Upon cisplatin injury, the morphology and arrangement of the residual cells changed. Previous studies have shown that cisplatin-induced hair cell damage increases from the apex to the base, which might be attributed to the lower levels of the antioxidant glutathione in basal hair cells compared to apical hair cells [77]. At a concentration of 10 μM, nuciferine preserved more hair cells in the apical turn but failed to protect additional hair cells in the middle and basal turns. Furthermore, 10 μM nuciferine improved the morphology and arrangement of hair cells in the apical and middle turns but had no significant effect on those in the basal turns (Figure 2). This suggests that 10 μM may be too low to exert protective effects on cochlear basal cells. In contrast, higher concentrations of nuciferine (30, 60, and 90 μM) exhibited better protective effects against cisplatin-induced ototoxicity in the basal turn (Figure 2). Among these, 60 μM nuciferine provided the most potent protective effect on hair cells, demonstrated by a more significant number and better-organized arrangement of residual hair cells, indicating it is the optimal protective concentration in vitro. Although 90 μM nuciferine also had a protective effect, it caused minor cytotoxicity due to the high dosage. 

Atoh1 labels hair cells and overlaps with hair cell-specific marker Myosin7a in cochlear explants [53]. As shown in Figure 3A, after using the FerroOrange probes, hair cells in each group (including the control group) were more dispersedly arranged, and the space between inner and outer hair cells increased. The subtle changes in hair cell morphology in these groups may be attributed to the mild toxicity of FerroOrange probes; however, this does not affect the observation of iron accumulation in cochlear hair cells. 

Natural active products have been reported to possess certain pharmacological bioactivities, and some of them have been proven to protect hair cells from damage caused by cisplatin [56,79,80]. Nuciferine exhibits anti-inflammatory, antioxidant, anti-obesity [48,81,82], and anti-cancer [83] activities. Nuciferine also can protect against acute kidney injury [52] and lipopolysaccharide (LPS)-induced endometritis [57] by inhibiting ferroptosis. In our study, nuciferine exhibited a noticeable anti-ferroptosis effect in both RSL3-induced and cisplatin-induced hair cell damage (Figure 1 and Figure 2). Nuciferine largely inhibited cisplatin-induced activation of ferritinophagy (Figure 4 and Figure 5), reduced the overload of ferrous iron (Figure 3), and thus reduced hair cell damage (Figure 2). Moreover, nuciferine reduced cochlear hair cell loss and ribbon synapse damage in an acute hearing loss mouse model (Figure 8). Importantly, nuciferine protected mouse hearing function. ABR measurement demonstrated an approximately 20 dB improvement in hearing threshold (Figure 7), suggesting that nuciferine is a promising therapeutic agent to protect against cisplatin-induced hearing loss.

Our findings provide evidence of an effective otoprotective agent, nuciferine, to protect from cisplatin-induced ototoxicity by inhibiting NCOA4-mediated ferritinophagy (Figure 3). However, how nuciferine suppresses NCOA4 expression remains to be further explored. Although nuciferine can rapidly cross the blood–brain barrier [84], its ability to cross the blood–labyrinth barrier needs to be further studied. In addition, the poor solubility of nuciferine in both water and DMSO limits its bioavailability and efficacy. Previous study has reported the structure of nuciferine, a tetracyclic core with two methoxy substituents, one methyl group connected to nitrogen, and one chirality center [85]. Modifying the structure or functional groups of nuciferine may help improve its solubility to acquire better therapeutic effects.

## 5. Conclusions

**Figure 9:**
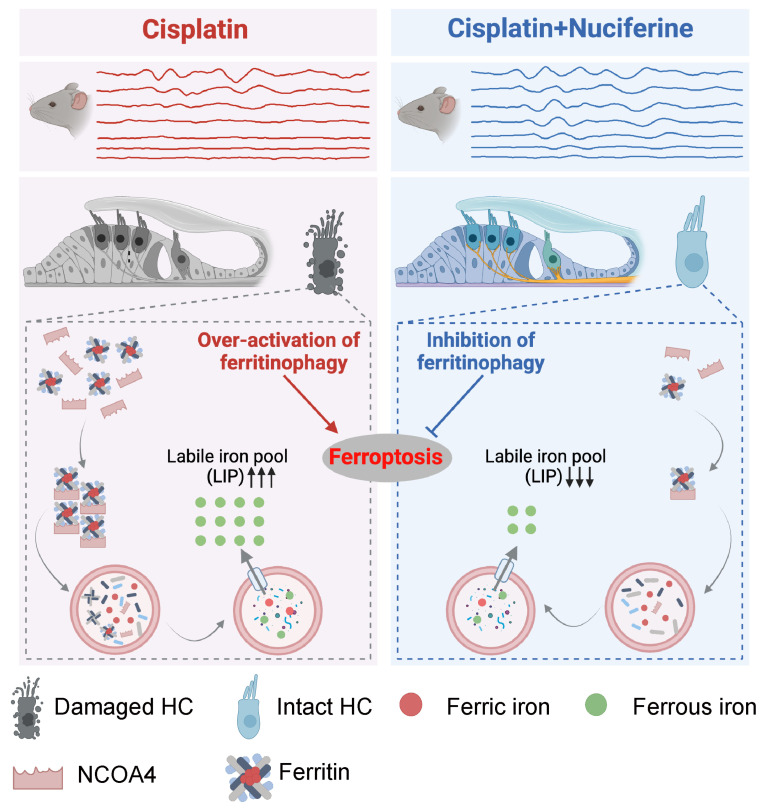
The possible mechanisms of nuciferine protective effects on cisplatin-induced ototoxicity. The left and right parts of the diagram represent cisplatin-injured hair cells and nuciferine co-treatment-rescued hair cells, respectively. After cisplatin treatment, the process of ferritinophagy was over-activated in the injured hair cells. The selective cargo receptor of ferritinophagy, NCOA4, was upregulated, which in turn recognized and delivered ferritin into lysosomes for complete degradation and ferric iron (Fe^3+^) release. The released lysosomal Fe^3+^ can be reduced to ferrous iron (Fe^2+^) in the lysosome and exported to the cell plasma, promoting the increase in the intracellular labile iron pool (LIP) and the lethal ferroptosis of cochlear hair cells. Nuciferine protected against cisplatin-induced ototoxicity by potently inhibiting ferritinophagy, reducing intracellular LIP, and regulating the iron homeostasis in hair cells. Therefore, nuciferine could be developed as a promising protective agent for treating cisplatin-induced hearing loss.

Overall, the current work shows that NCOA4-mediated ferritinophagy is involved in cisplatin-induced ototoxicity, and nuciferine can inhibit cisplatin-induced ferroptosis of cochlear hair cells by regulating ferritinophagy and iron homeostasis. Nuciferine alleviated cisplatin-induced ototoxicity and improved hearing function in both in vitro and in vivo experiments (Figure 9). These results suggest that the inhibition of ferritinophagy is an important therapeutic target and nuciferine is a promising natural agent for treating cisplatin-induced hearing loss.

## Figures and Tables

**Table 1:** The primers designed for the quantitative real-time PCR.

Assay	Target Gene	Sequence	Primer	Accession Number	Product Length
1	*Ptgs2*	GCTTCAGGAGTCAGTCAGGA	Forward	NM_011198.5	188
		ACGGTTTTGACATGGATTGGA	Reverse		
2	*Aifm2*	GGAGTACATCAAGGTGGAGAC	Forward	NM_001039194.3	109
		CTCAAATGCACTGCGGTAGG	Reverse		
3	*Gpx4*	AACAGCTCCGAGTTCCTGG	Forward	NM_001037741.4	131
		CACACGAAACCCCTGTACTT	Reverse		
4	*Nrf2*	GCAGGACATGGATTTGATTGAC	Forward	NM_010902.5	223
		CGGCTGAATTGGGAGGAATT	Reverse		
5	*Slc7a11*	ATCATCACAGTGGGCTACGT	Forward	NM_011990.2	122
		GAGAATTTTCCCAGCAGCCG	Reverse		
6	*Acsl4*	AGCGTTCCTCCAAGTAGACC	Forward	NM_001033600.1	159
		GTCCTTCGGTCCTAGTCCAG	Reverse		
7	*Lpcat3*	TTGACTACTACGATGGAGGCA	Forward	NM_145130.3	150
		GGTTCATTGAAAATTGGGGCC	Reverse		
8	*Alox15*	CCTGGATCTTCTCAAGCAAGC	Forward	NM_009660.3	159
		ATTCCCACCACGTACCGATT	Reverse		
9	*Irp1*	TTGGAGCCAAGCAAGGATTT	Forward	NM_007386.3	156
		CGGATGGATTGCTGGTGTTT	Reverse		
10	*Ireb2*	GCATCCCAGCCTATTGAGAATG	Forward	NM_022655.4	105
		AGCAGCACTACTCCTAGCAATA	Reverse		
11	*Dmt1*	TACAGTGAAGCCCAGCCAGA	Forward	NM_001146161.1	114
		ATGATCACAGCTCCCACGAT	Reverse		
12	*Slc40a1*	CCAGATTATGACATTTGGCTCC	Forward	NM_016917.2	199
		AACCTTCCAGAGCAGAACGT	Reverse		
13	*Trf*	CGCAGTCCTCTTGAGAAAGC	Forward	NM_133977	172
		CACCGCCATCTTTCAGACAC	Reverse		
14	*Tfrc*	TGATTGGATTCATGAGTGGCT	Forward	NM_001357298.1	153
		GGTCTGCCCAATATAAGCGAG	Reverse		
15	*Fth1*	GACCGTGATGACTGGGAGAG	Forward	NM_010239.2	100
		TAGCCAGTTTGTGCAGTTCC	Reverse		
16	*Ftl*	CAGCCTGGTCAATTTGTACCT	Forward	NM_010240.2	114
		GCCAATTCGCGGAAGAAGTG	Reverse		
17	*Ncoa4*	AGCAGAAGTCAGCATCCAGT	Forward	NM_001033988.3	112
		AGTCCTGTGGGTTGGTACTG	Reverse		
18	*Sqstm1*	ACCCACTACCCCAGAAAGTT	Forward	NM_011018.3	157
		CACCGACTCCAAGGCTATCT	Reverse		
19	*Gapdh*	TCATCATCTCCGCCCCTTC	Forward	NM_001411843.1	170
		CATGAGCCCTTCCACAATGC	Reverse		

## Data Availability

The data presented in this study are available on request from the corresponding author.

## Supplementary Materials

The following supporting information can be downloaded at: https://www.mdpi.com/article/10.3390/antiox13060714/s1.
