# Supplementary material for: Nuciferine Protects Cochlear Hair Cells from Ferroptosis through Inhibiting NCOA4-Mediated Ferritinophagy
Source: Antioxidants (Basel). 2024 Jun 12;13(6):714. doi: 10.3390/antiox13060714 (PMC11201048; doi:10.3390/antiox13060714)
Supplement: Supplementary file 1 [file antioxidants-13-00714-s001.zip › antioxidants-2993696-supplementary.pdf]

Supplementary materials

1 **Nucleic acid extraction:** The RNA extraction protocol comes from Invitrogen. All procedures were carried out on the clean experimental bench. The experimenter wore a mask, gloves and isolation suit to avoid the degradation of RNA. The concentration and purity of RNA were assessed by NanoDrop2000 spectrophotometer (Thermo Scientific, Massachusetts, America). When the concentration of a sample is between 100 and 200 ng/μl, the OD260/OD280 value is between 1.8 and 2.0, and the OD260/OD230 value is between 1.6 and 2.2, the sample is considered qualified. RNA integrity was assessed by gel electrophoresis. The following part is the detailed protocol for RNA extraction.

RNA extraction protocol

A. Preparation:

1) RNase-free reagent: isopropanol; 75% ethanol (pre-cool to 4℃); RNase-free water; Chloroform; Trizol (Invitrogen, 15596026CN).
2) Pre-cool the refrigerated highspeed centrifuge 5810R (Eppendorf, Hamburg, Germany) to 4℃.
3) 10 μl, 100 μl and 1000 μl RNase-free pipette tips; 1.5 ml and 200 μl RNase-free microtubes.

B. Extraction protocol

1) Lyse samples and separate phases

- Add 1 ml of TRIzol™ Reagent per 8 cochleae for lysis.
- Pipet the lysate up and down several times to homogenise.
- Incubate for 5 minutes to allow complete dissociation.
- Add 200 μl of chloroform per 1 ml of TRIzol™ Reagent used for lysis, securely cap the tube, then thoroughly mix by shaking.
- Incubate for 2–3 minutes.
- Centrifuge the sample for 15 minutes at 12,000 × *g* at 4°C.
  The mixture separates into a lower phenol-chloroform, an interphase, and a colorless upper aqueous phase.
- Transfer 400 μl of the aqueous phase containing the RNA to a new tube.

2) Precipitate the RNA

- Add 500 μl of isopropanol to the above aqueous phase per 1 ml of TRIzol™ Reagent used for lysis.
- Incubate for 10 minutes at 4°C.
- Centrifuge for 10 minutes at 12,000 × g at 4°C. Total RNA precipitate forms a white gel-like pellet at the bottom of the tube.
- Discard the supernatant with a micropipette.

3) Wash the RNA

- Resuspend the pellet in 1 mL of 75% ethanol per 1 mL of TRIzol™ Reagent used for lysis.
- Vortex the sample briefly, then centrifuge for 5 minutes at 7500 × g at 4°C.
- Discard the supernatant with a micropipette.
- Vacuum or air dry the RNA pellet for 5–10 minutes.

4) Solubilize the RNA

- Resuspend the pellet in 20 μl of RNase-free water by pipetting up and down.

2 **Reverse transcription:** 2 μg of sample RNA was reverse transcribed into cDNA using PrimeScript™ RT reagent Kit with gDNA Eraser (Takara, RR047A). The protocol was provided as follows:

1) Step 1: Genomic DNA Removal Reaction

Reagent name	Volume（20 μl）
5*gDNA Eraser Buffer	4 μl
gDNA Eraser	2 μl
Sample RNA	14 μl

Reaction condition : 42℃ 2min，4℃;

2) Step 2: Reverse transcription reaction:

Reagent name	Volume (40 μl)
Reaction solution from step 1	20 μl
PrimeScript RT Enzyme Mix I	2 μl
RT Primer Mix	2 μl
5×PrimeScript Buffer 2	8 μl
RNase Free dH2O	8 μl
Total	40 μl

Reaction condition: 37℃ 15min, 85℃ 5sec, 4℃.

3 **qPCR protocol:**

1) Reaction system configuration：

Reaction system	Volume
TB Green Premix Ex Taq II (2*)	10 μl
PCR Forward Primer (10μM)	1 μl
PCR Reverse Primer (10μM)	1 μl
ROX Reference Dye II (50X)	0.4 μl
cDNA	1 μl
ddH2O	6.6 μl

2) Reaction Condition:

Stage 1：Pre-denaturation	95°C 30s
Stage 2：PCR reaction	95°C 5s
	60°C 30s
	Reps：40
Stage 3 : Melt curve	95°C 15s
	60°C 60s
	95°C 15s
